# Retraction Note: Growth inhibition and apoptosis in colorectal cancer cells induced by vitamin D-Nanoemulsion (NVD): involvement of Wnt/β-catenin and other signal transduction pathways

**DOI:** 10.1186/s13578-024-01262-0

**Published:** 2024-06-08

**Authors:** Suhail Razak, Tayyaba Afsar, Ali Almajwal, Iftikhar Alam, Sarwat Jahan

**Affiliations:** 1 Department of Animal Sciences, Faculty of Biological Sciences, Quaid-i-Azam University, Islamabad, Pakistan; 2 Department of Community Health Sciences, College of Applied Medical Sciences, King Saud University, Riyadh, Kingdom of Saudi Arabia

**Retraction Note: Cell Biosci (2019) 9:15**

10.1186/s13578-019-0277-z

The Editor-in-Chief has retracted this article. After publication, concerns were raised regarding the validity of the results presented. Following an investigation by the publisher, numerous issues were highlighted, including, but not limited to:

- In Fig. 4a, the following blot images appear highly similar to the corresponding blot images (except where noted) in Fig. 4a of [1].
  - Cyclin A.
  - Cyclin B1.
  - Cyclin E2.
  - P-cdc2(Tyr15).
  - Cdk-2 (P-cdc25c / cdc25c in [1])
  - Cdk-4 (P-Myt1 / Interphase Myt1 in [1])
  - P-P21 / P21(waf1/cip1).
- In Fig. 4c, the blot images appear highly similar to the blot images in Fig. 5a of [1].
- In Fig. 4d, the Beta-Actin blot image appears highly similar to the Fig. 10a Beta-Actin blot image of [2].
- In Fig. 5a, the Beta-Actin blot image appears highly similar to the Fig. 5b Beta-Actin blot image of [1].
- In Fig. 5a, the Bak blot image appears highly similar to the Fig. 4a Cyclin B1 blot image of [1].
- In Fig. 7a, the Control (HCT116) image appears highly similar to the Fig. 7a Control (HCT116) of [2].
- In Fig. 7, the images 7a, 7b, 7c, and 7d appear to overlap with the Fig. 5B 22Rv1 Control and MEM panels of [3].
- In Fig. 8a, the Beta-Actin blot image appears highly similar to the Fig. 9c Beta-Actin blot image of [2].
- In Fig. 9b, the Control image appears highly similar to the Fig. 9b Control of [2].
- In Fig. 10a, the Caspase3 blot image appears highly similar to the Fig. 5A Procaspase 7 / Cleaved Caspase 7 blot image of [1].
- In Fig. 12a, the Beta-Actin blot image appears highly similar to the Fig. 4A Beta-Actin blot of [1].
- In Fig. 12a, the AKT blot image appears highly similar to the Fig. 4A P-Myt1 / Interphase Myt1 blot of [1].
- In Fig S1, the panels appear highly similar to the Fig. 7B and 7C panels of [3].

In addition, the article contains text that substantially overlaps with [2].

The authors have not responded to correspondence from the Publisher about this retraction.
